# Lockdown Impact on Stress, Coping Strategies, and Substance Use in Teenagers

**DOI:** 10.3389/fpsyt.2021.790704

**Published:** 2022-01-21

**Authors:** Cédrine Bourduge, Frédérique Teissedre, Florence Morel, Valentin Flaudias, Marie Izaute, Georges Brousse

**Affiliations:** ^1^LAPSCO, CNRS, Université Clermont Auvergne, Clermont-Ferrand, France; ^2^Service d'addictologie et Pathologies Duelles, Centre Hospitalier Universitaire de Clermont-Ferrand, Clermont-Ferrand, France; ^3^CNRS, Institut Pascal, Université Clermont Auvergne, Clermont Auvergne INP, Clermont-Ferrand, France; ^4^Laboratoire de Psychologie des Pays de la Loire (LPPL-EA-4638), Université de Nantes, Univ Angers, Nantes, France

**Keywords:** COVID-19, lockdown, teenagers, stress level, coping strategies, substance use

## Abstract

**Background:**

In response to the COVID-19 pandemic, the French government took many measures, the most notable of which was a national lockdown on 17 March 2020. Its effects have been widely studied, but to our knowledge, no study has sought to determine how adolescents have adapted to cope with this situation. The present study set out to explore teenagers' stress levels, coping strategies, and substance use during this period.

**Methods:**

This paper is a cross-sectional study that rides on an existing prevention program interviewed 348 French middle school students (209 girls and 139 boys) in grade 8 (*M*_age_ = 13.45; SDage = 0.54) using an online questionnaire between March 17 and May 11, 2020 (COVID-19 lockdown). The study examined the teenagers' perceived stress, coping strategies they had used, including recent use of tobacco, alcohol and cannabis, during COVID-19 lockdown.

**Results:**

Teenagers reported lower perceived stress during lockdown than usually, with a significant decrease for girls. Those who perceived the least social support reported the highest levels of stress. The strategies of planning, behavioral disengagement, self-distraction, positive reframing, acceptance, and religion were used more than usual, while active coping and self-blame were used less. Acceptance was the most often used strategy and a source of decreased stress during lockdown. A significant decrease in recent tobacco, alcohol and cannabis use was also observed.

**Conclusion:**

Changes in the use of coping strategies, withdrawal from the stressful school environment, and greater exposure to parents than to peers caused adolescents to be less stressed and to decrease their substance use during the lockdown.

## Introduction

The COVID-19 pandemic began in China, in the Wuhan region, in December 2019, and later spread to Europe. The first cases reached France in late January 2020. The French government then implemented many measures, the most notable of which was a national lockdown on March 17, 2020, for a period of 2 months. Among children and adolescents, the prolonged closure of schools, involving disruption of educational, sports and social activities, coupled with home lockdown, may have had negative effects on their physical and psychological health (1). However, to our knowledge, no studies have investigated how adolescents adapted to cope with this novel situation. The present study examined the stress levels, coping strategies, and substance use of teenagers in this context.

Hawryluck et al. (2) previously highlighted that quarantine beyond 10 days in a pandemic setting increased stress. In addition, the fear of being infected or of infecting others, isolation (3), intolerance to uncertainty (4), cessation of work activities (5), or exposure to conflicting information from the media (6) are also important factors in increasing stress. This increase in stress was indeed found in adults (1, 5, 7), in children and in teenagers (3, 8, 9) during the COVID-19 pandemic lockdown. This stressful situation is a factor that could influence the mental health of adolescent (10, 11), because they are more vulnerable than adults to mental health problems, in particular during a lockdown (12). However, the literature shows that social support is a factor in decreasing symptoms in the face of stressful events (13–15). A study showed that prisoners in solitary lockdown had more depressive and anxious symptoms than those in non-solitary lockdown (16). During lockdown, those most stressed were those who received the least social support (7).

In this stressful context of lockdown, the coping strategies mobilized by each person may explain the inter-individual differences observed during this period. Coping is defined as “the cognitive and behavioral efforts made to master, tolerate, or reduce external and internal demands and conflicts among them” [(17), p. 223]. Coping is evolutionary: it adapts to each stressful event to reduce the effect of stress on well-being (18). Coping therefore depends on people's ability to develop new strategies and to abandon those that have become ineffective (19–21). This adaptive capacity appears as early as mid-childhood, with the development of metacognitive abilities that allow better adjustment of coping efforts to the stressor through an increase in the diversity and flexibility of available coping responses (20). During the first half of adolescence (grades 6–8), planning, positive reframing and acceptance strategies tend to be emphasized (22). Strategies related to emotional and instrumental support begin to be used in the second half of adolescence (grade 9–12). The most functional strategies, i.e., those that act most effectively on the stressor, are active coping, planning, positive reframing and acceptance. By contrast, denial, behavioral disengagement, and substance use are dysfunctional (23–26).

During lockdown, studies have still found increased tobacco and alcohol use in the general population (27–29). However, teenagers use these substances differently from adults: it is during adolescence that substance use behaviors begin, become established and cause developmental and mental health disorders (30). At this age, alcohol is the most often consumed product, followed by tobacco, and finally various other drugs (31). Two psychosocial factors come into play as a “pattern” of vulnerability to substance use: parental and peer influence (32). For Windle (33), parents are an important protective factor against substance use. However, after the age of 12, parental influence decreases, while peer influence increases (34). It is peers who encourage experimentation (35): they provide direct access to substances and socially reinforce their use (36). Hence in adolescence, substance use takes place within the peer group, not in the family sphere (29). However, during lockdown, teenagers remained in the family home, limiting exchanges with peers to virtual contact, and so likely reducing their influence on substance use behavior.

In the context of the first COVID-19 pandemic lockdown, the present study examined the stress level, coping strategies and substance use of teenagers. In the light of recent studies, we hypothesized an increase in stress levels. This variation would be sensitive to differences in perceived social support, classically observed in the literature. We also expected a modification in the coping strategies mobilized during lockdown, along with a change in their effectiveness on stress. Finally, we expected that teenagers would decrease their use of tobacco, alcohol and cannabis.

## Methods

### Participants

**Figure 1:**
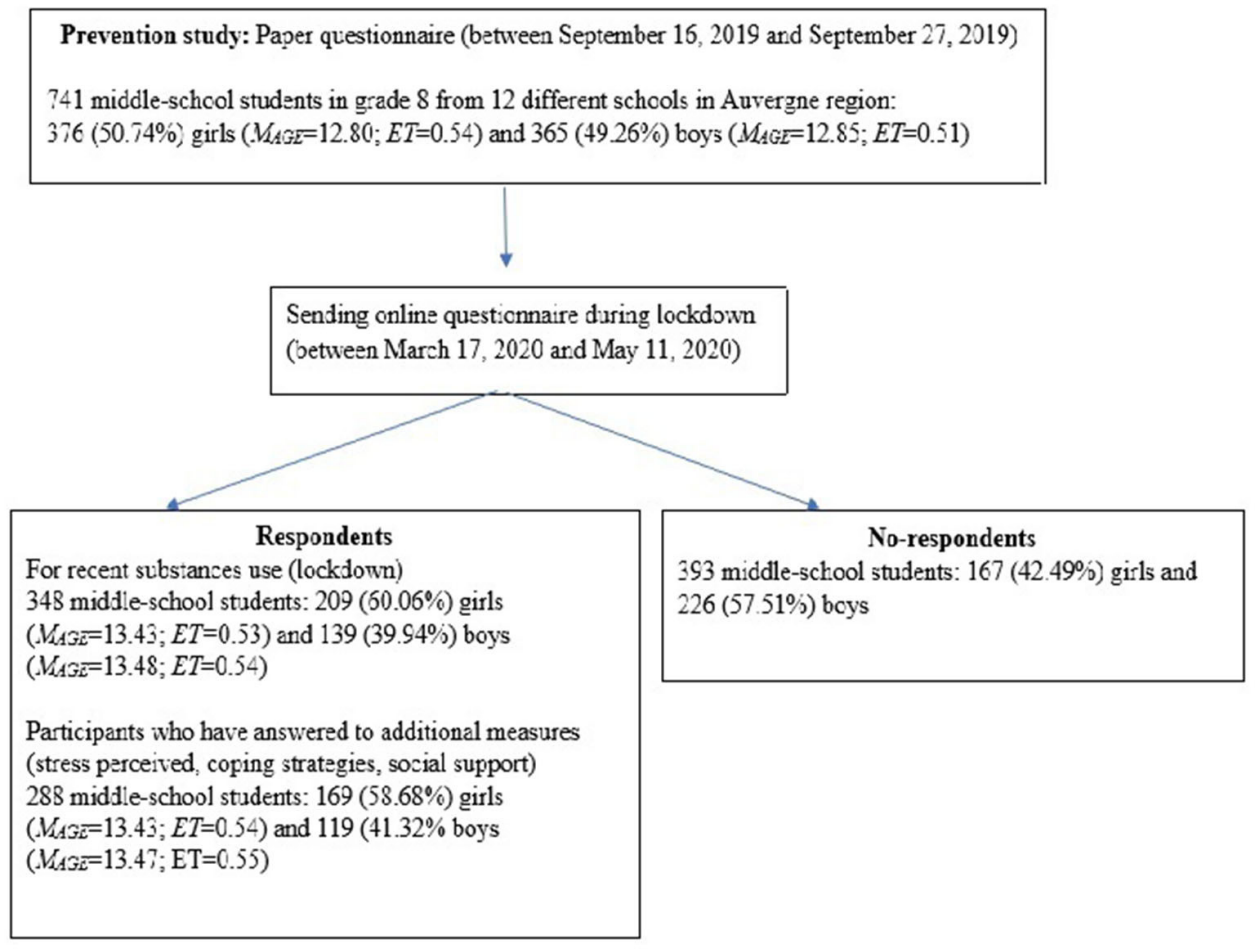
Flowchart of the inclusion procedure.

Three hundred and forty-eight middle school students [209 (60.06%) girls and 139 (39.94%) boys] in grade 8 (*M*_age_ = 13.45, SD_age_ = 0.54) from 12 schools in the Auvergne-Rhône-Alpes region of France took part in the study. Initially, these participants were part of a voluntary sample (741 middle-school students, see Figure 1) to test an addiction prevention program within their school during the school year (37), based on self-concept theory (Bourduge et al., in prep). Schools have voluntarily chosen to participate in the prevention program. Students were only able to participate with parental consent. This study was conducted in accordance with ethical standards and has the approval of local ethics comities (INSERM agreement reference: 19||134-00, ANSM registration number: 2019-A03131-56).

### Materials and Procedure

The prevention program consisted of 13 1-h interactive sessions and was based on Social Influence approach and addresses social and personal skills, knowledge, and normative beliefs. In order to evaluate this program, a paper questionnaire of the recent tobacco, alcohol and cannabis use (use of the product at least once during the 30 days preceding the survey) was completed at the beginning of the school year (October 2019) (see Figure 1).

Then, an online questionnaire was sent by the schools to the students during the lockdown (between March 17, 2020, and May 11, 2020) using a link generated by the Qualtrics XM online questionnaire creation software. Three hundred and forty-eight participants (see Figure 1) then answered questions about recent tobacco, alcohol and cannabis use. Additional measures were added to assess the impact of lockdown. The perceived stress level was measured usually and during lockdown. Only one question measured, from 1 (“Not stressed at all”) to 10 (Extremely stressed”), the stress level usually (“Are you usually a stressed person?”) and during lockdown (“How were you stressed during lockdown?”). A high score indicates a high level of stress. Coping strategies used were measured with the French version (23) of the Brief-COPE (38). The Brief COPE contains 28 items assessing the following coping dimensions: active coping, planning, use of instrumental support, use of emotional support, venting, behavioral disengagement, self-distraction, self-blame, positive reframing, humor, denial, acceptance, religion and substance use. Each of the 14 dimensions was measured with the sum of 2 items, scored with a 4 point-scale ranging from 1 (“Not at all”) to 4 (“always”). A high score indicates a strategy estimated to be used a lot. We used the scale in the dispositional format to assesses how teenagers cope usually (active coping α = 0.34, planning α = 0.58, use of instrumental support α = 0.78, use of emotional support α = 0.77, venting 0.55, behavioral disengagement α = 0.59, self-distraction α = 0.30, self-blame α = 0.68, positive reframing α = 0.69, humor α = 0.70, denial α = 0.58, acceptance α = 0.70, religion α = 0.74, and substance use α = 0.53) and the situational format to assesses how teenagers cope during lockdown (active coping α = 0.41, planning α = 0.63, use of instrumental support α = 0.77, use of emotional support α = 0.78, venting 0.62, behavioral disengagement α = 0.58, self-distraction α = 0.37, self-blame α = 0.51, positive reframing α = 0.71, humor α = 0.71, denial α = 0.58, acceptance α = 0.70, religion α = 0.71, and substance use α = 0.71). Perceived social support was measured through 3 elements. Staying in contact with their friends and how much they missed them was measured with only one question each, on a 10 point-scale from 1 (“Not at all”) to 10 (“A lot”). A high score indicates that they stayed a lot in contact with their friends or that they missed their friends a lot. How many hours they spent online per day with their friends was measured with a slider from 0 to 24 h. The higher the number, the more time the participants spent online each day with their friends (see Supplementary Material for details). However, owing to the time required and the large number of scales, only 288 participants completed the additional measures (see Figure 1).

### Statistical Analyses

**Figure 2:**
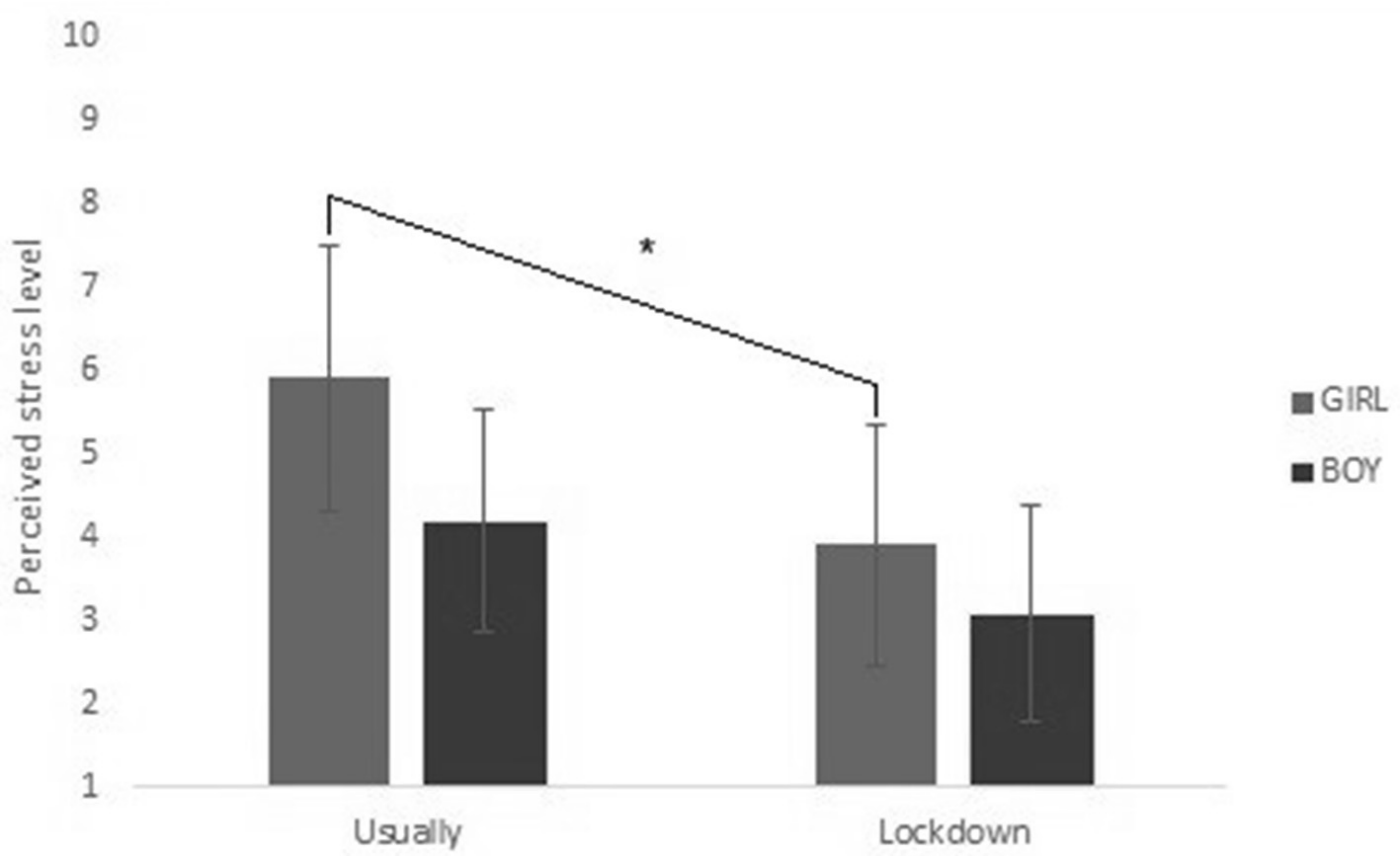
Interaction effect between lockdown and gender in stress. *The significative effect described in the section results, in stress paragraph.

The analyses of this study were performed using SPSS 25 software. We have ensured the normality of our data. The effect of lockdown (IV) (difference between “usually” and “during lockdown”) and gender (IV) on stress level (DV) (see Figure 2) was measured using a repeated measures ANOVA test. The impact of perceived social support (IVs) (staying in contact, missing, and time online) on stress level (DV) was measured using multiple linear regressions.

The impact of lockdown (IV) and gender (IV) on the estimated use of coping strategies (DV) was measured using a repeated measures ANOVA test (see Table 1). The effect of coping strategies (IV) on stress levels (DV) was measured using multiple linear regressions.

**Table 1:** Estimate of coping strategies used usually and during lockdown, by gender.

**Brief-COPE**	**Usually**			**Lockdown**			**Lockdown** **effect** ***p***	**Gender** **effect** ***p***	**Lockdown** **× gender** ***p***
	**Total** ***M* (SD)**	**Girls** ***M* (SD)**	**Boys** ***M* (SD)**	**Total** ***M* (SD)**	**Girls** ***M* (SD)**	**Boys** ***M* (SD)**			
Active coping	4.45 (1.43)	4.42 (1.39)	4.50 (1.48)	4.28 (1.49)	4.21 (1.44)	4.38 (1.55)	<0.05^*^	>0.05	>0.05
Planning	4.19 (1.56)	4.27 (1.55)	4.08 (1.57)	4.34 (1.76)	4.40 (1.81)	4.26 (1.69)	<0.05^*^	>0.05	>0.05
Using instrumental support	4.20 (1.66)	4.40 (1.69)	3.91 (1.57)	4.08 (1.75)	4.20 (1.81)	3.90 (1.61)	>0.05	<0.05^*^	>0.05
Using emotional support	3.86 (1.65)	4.05 (1.77)	3.58 (1.43)	3.88 (1.80)	4.04 (1.88)	3.67 (1.67)	>0.05	<0.05^*^	>0.05
Venting	3.86 (1.61)	3.97 (1.65)	3.72 (1.54)	3.85 (1.70)	3.93 (1.77)	3.74 (1.61)	>0.05	>0.05	>0.05
Behavioral disengagement	3.17 (1.39)	3.13 (1.43)	3.23 (1.35)	3.38 (1.49)	3.45 (1.54)	3.30 (1.40)	<0.01^**^	>0.05	>0.05
Self-distraction	5.07 (1.45)	5.22 (1.34)	4.86 (1.58)	5.37 (1.57)	5.51 (1.43)	5.18 (1.74)	<0.001^***^	<0.05^*^	>0.05
Self-blame	4.16 (1.74)	4.49 (1.79)	3.70 (1.56)	3.95 (1.65)	4.21 (1.64)	3.58 (1.51)	<0.01^**^	<0.001^***^	>0.05
Positive reframing	4.74 (1.72)	4.87 (1.75)	4.56 (1.67)	4.87 (1.83)	4.99 (1.88)	4.70 (1.74)	<0.05^*^	>0.05	>0.05
Humor	4.15 (1.73)	4.05 (1.73)	4.27 (1.73)	4.07 (1.83)	3.98 (1.90)	4.20 (1.73)	>0.05	>0.05	>0.05
Denial	3.10 (1.42)	3.27 (1.54)	2.86 (1.20)	3.09 (1.42)	3.16 (1.49)	3.02 (1.30)	>0.05	>0.05	<0.05^*^
Acceptance	5.74 (1.72)	5.65 (1.73)	5.85 (1.72)	5.91 (1.74)	5.88 (1.70)	5.96 (1.79)	<0.01^**^	>0.05	>0.05
Religion	2.44 (1.06)	2.46 (1.06)	2.40 (1.06)	2.50 (1.17)	2.53 (1.18)	2.46 (1.16)	<0.05^*^	>0.05	>0.05
Substance use	2.10 (0.50)	2.09 (0.42)	2.11 (0.54)	2.10 (0.48)	2.07 (0.34)	2.13 (0.62)	>0.05	>0.05	>0.05

* *p < 0.05,*

** *p < 0.01,*

*** *p < 0.001*.

**Figure 3:**
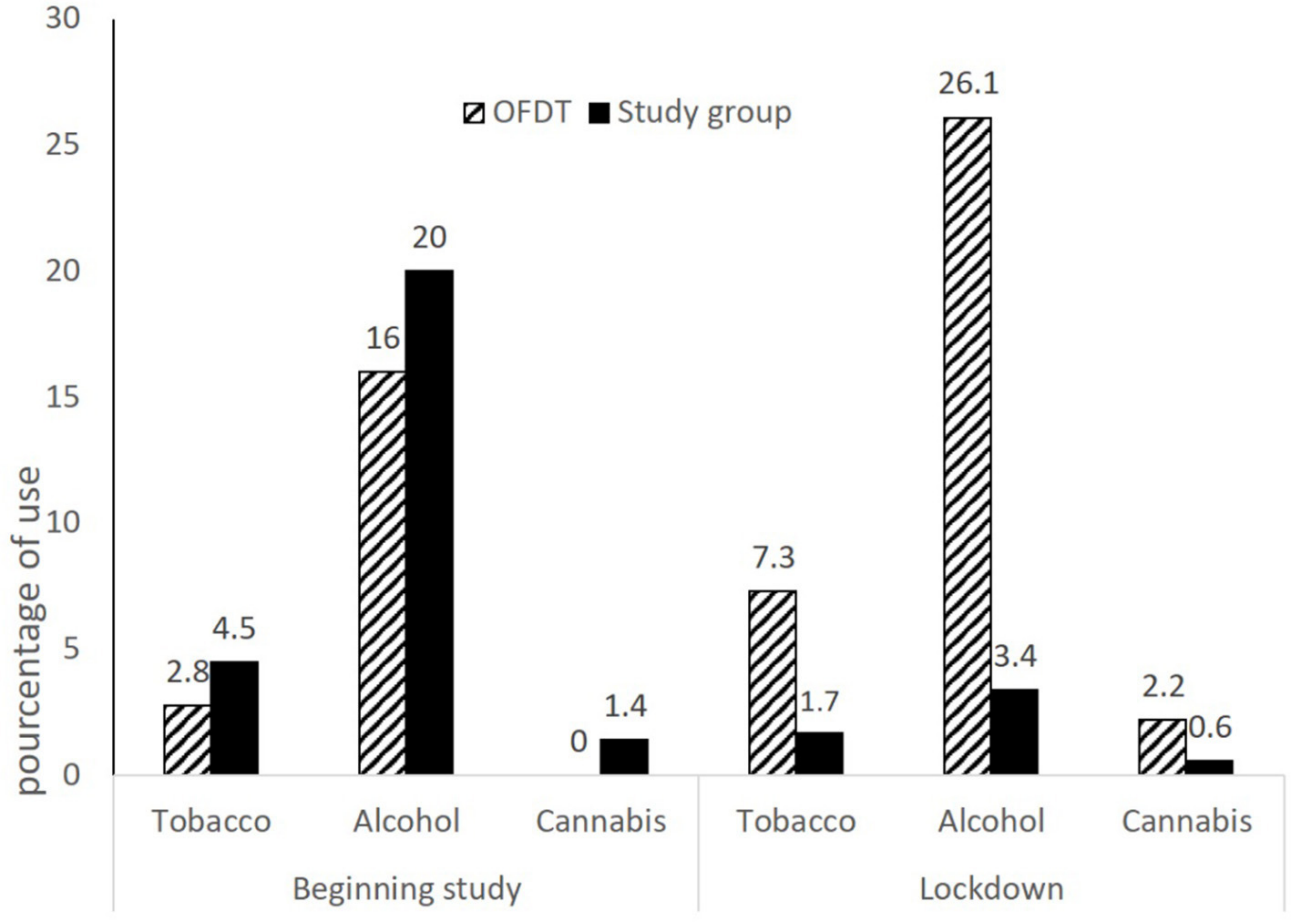
Recent tobacco, alcohol and cannabis use at the beginning of the school year and during lockdown, compared with data collected by OFDT.

The numbers of tobacco, alcohol and cannabis use at baseline and during lockdown were compared with the French Drug Observatory (OFDT) data (31) (see Figure 3). The OFDT is a French organization which collects national substance use data every 4 years. This comparison was made using a confidence interval calculated on our data: we looked to see whether the OFDT data fell within this interval. No other factors could be taken into account because of the small number of consumers.

## Results

### Stress

Our participants mostly felt less stressed [*F*_(1, 286)_ = 70.01, *p* < 0.001,$\eta_{p}^{2}$= 0.197] during the lockdown (*M* = 3.57, SD = 2.82) than they usually do (*M* = 5.21, SD = 3.10). 56.90% felt less stress, 22.90% felt the same stress, and 20.10% felt more stress during lockdown.

We also observed a main effect of gender [*F*_(1, 286)_ = 19.168, *p* < 0.001, $\eta_{p}^{2}$= 0.063], with higher perceived stress in girls than in boys. In addition, the interaction effect between lockdown and gender [*F*_(1, 286)_ = 5.661, *p* < 0.05, $\eta_{p}^{2}$= 0.019] revealed a greater decrease in stress for girls than for boys during lockdown (see Figure 2).

We could also see that during lockdown, the more they missed their friends [*B* = 0.369, *t*_(145)_ = 4.095, *p* < 0.01], the higher was their stress level. And the more they stayed in contact with them [*B* = −0.210, *t*_(145)_ = −2.240, *p* < 0.05], the lower was their stress level. Nevertheless, the time spent per day online with their friends did not influence their stress level [*B* = −0.044, *t*_(145)_ = −0.539, *p* > 0.05] [*R*^2^= 0.108, *F*_(3, 145)_ = 5.831; *p* < 0.01].

### Coping Strategies

Coping data are reported in Table 1. Usually and during lockdown, acceptance, self-distraction and positive reframing strategies are estimated to be the most often used during stressful situations. Conversely, religion and substance use are estimated to be the least often used. During lockdown, our participants estimated they had significantly increased the use of planning [*F*_(1, 279)_ = 4.134, *p* < 0.05, $\eta_{p}^{2}$ = 0.015], behavioral disengagement [*F*_(1, 279)_ = 8.552, *p* < 0.01, $\eta_{p}^{2}$ = 0.030], self-distraction [*F*_(1, 279)_ = 18.275, *p* < 0.001, $\eta_{p}^{2}$ = 0.061], positive reframing [*F*_(1, 279)_ = 4.427, *p* < 0.05, $\eta_{p}^{2}$= 0.016], acceptance [*F*_(1, 279)_ = 7.341, *p* < 0.01, $\eta_{p}^{2}$= 0.026] and religion [*F*_(1, 279)_ = 5.806, *p* < 0.05, $\eta_{p}^{2}$ = 0.020]. On the contrary, active coping [*F*_(1, 279)_ = 5.449, *p* < 0.05, $\eta_{p}^{2}$ = 0.019] and self-blame [*F*_(1, 279)_ = 10.326, *p* = 0.001, $\eta_{p}^{2}$ = 0.036] were estimated to be less often used.

Girls estimated using instrumental [*F*_(1, 279)_ = 3.979, *p* < 0.05, $\eta_{p}^{2}$ = 0.014] and emotional [*F*_(1, 279)_ = 4.566, *p* < 0.05, $\eta_{p}^{2}$ = 0.016] support, self-distraction [*F*_(1, 279)_ = 4.118, *p* < 0.05, $\eta_{p}^{2}$ = 0.015] and self-blame [*F*_(1, 279)_ = 13.652, *p* < 0.001, $\eta_{p}^{2}$ = 0.047] more than boys. Finally, we found an interaction effect between lockdown and gender for denial [*F*_(1, 279)_ = 4.499, *p* < 0.05, $\eta_{p}^{2}$ = 0.016]. Girls felt they used this strategy less during lockdown than usually, but boys felt they used it more.

### Coping Strategies and Stress

Usually, the estimate of self-blame use [*B* = 0.270, *t*_(266)_ = 4.262, *p* < 0.01] predicted increased stress. Estimating the use of active coping [*B* = −0.129; *t*_(266)_ = −2.041, *p* < 0.05], acceptance [*B* = −0.180, *t*_(266)_ = −2.681, *p* < 0.01] and substance use [*B* = −0.151, *t*_(266)_ = −2.630, *p* < 0.01] predicted decreased stress [*R*^2^= 0.236, *F*_(14, 266)_ = 5.885; *p* < 0.001].

During lockdown, the estimated use of emotional support [*B* = 0.367, *t*_(266)_ = 4.951, *p* < 0.01] and self-blame [*B* = 0.123, *t*_(266)_ = 2.091, *p* < 0.05] predicted increased stress. Estimated use of acceptance [*B* = −0.134, *t*_(266)_ = −2.064, *p* < 0.05] predicted decreased stress [*R*^2^ = 0.311, *F*_(14, 266)_ = 8.559; *p* < 0.001].

### Substance Use

The OFDT data (end of grade 7) for tobacco (2.80%), alcohol (16.00%) and cannabis (0.00%) were below the confidence intervals [CI_tobacco_ (3.21; 6.28), CI_alcohol_ (17.21; 23.11), CI_cannabis_ (0.76; 2.56)] of our data (beginning of grade 8). During lockdown, this observation was reversed: the OFDT data (end of grade 8) for tobacco (7.30%), alcohol (26.1%) and cannabis (2.20%) were found to be higher than our confidence intervals [CI_tobacco_ (0.79; 3.71), CI_alcohol_ (1.98; 5.93), CI_cannabis_ (0.16; 2.07)] (end of grade 8). This means that our figures were significantly lower than the ODFT data during lockdown, whereas at the beginning of the year they were significantly higher than the OFDT data (see Figure 3).

## Discussion

First, during lockdown, we observed a decrease in perceived stress. We also noted an evolution in the estimation of the use of coping strategies during lockdown, with in particular, a strong decrease in recent tobacco, alcohol and cannabis use.

Recently, studies have highlighted the deleterious impact of lockdown on stress in adults 1,2,4,5,7. For teenagers, on the contrary, we found a decrease in perceived stress during the COVID-19 pandemic lockdown. This decrease appears to be essentially explained by the fact that teenagers were less exposed to school pressures during this period through home-based learning. School, with teachers and pears pressure, marks or bullying, has been shown to be a stressful environment for teenagers (39, 40). This decrease was greater for girls than for boys, although they maintained higher levels of stress than boys. Girls tend to feel more affected and stressed by the school setting (40–43) and by teacher pressure (44) than boys. The fact that they are more stressed by the school setting explains why being removed from it had a greater impact on their stress level than on that of the boys. Finally, we found that 10.80% of the observed variance in stress could be explained by perceived social support. Consistent with the literature, those who perceived the least social support (7, 45, 46) had the highest levels of stress.

On the other hand, more than 30% of the differences in stress was also due to the coping strategies used by the teenagers. We noted that the use of acceptance and positive reframing strategies was favored, as classically observed during the first half of adolescence (22). We also found a gender difference, with greater use of instrumental support, emotional support and self-blame in girls than in boys (23). In addition, our participants altered their use of certain strategies to cope with lockdown (17–21). They increased their use of planning, behavioral disengagement, self-distraction, positive reframing, acceptance, and religion strategies during lockdown compared to usual, and decreased their use of active coping and self-blame. Finally, active coping and acceptance did explain a decrease in usual stress in our study, as noted in the literature in adults (23). However, during the lockdown situation, only acceptance explained the decrease in stress. In summary, during lockdown, a modification of the strategies mobilized could be observed. Acceptance was the most often used strategy and was a source of stress reduction. These findings could therefore also explain part of the decrease in stress observed in the teenagers during this period.

Among the coping strategies, substance use was estimated to be the least often used by teenagers, and we found no change in its use during lockdown. However, our results showed a decrease in recent tobacco, alcohol and cannabis use during lockdown, whereas at this age, use increases (31). We can hypothesize that during lockdown, teenagers remained in contact with their parents, who are generally considered a protective factor against substance use (33). By contrast, they had little exposure to the influence of their peers, with whom use at this age takes place (29), together with the first experimentation (35). We consider that this change in exposure to parents and peers would explain this decrease.

We identified several limitations to our study. First of all, the use of an online questionnaire, with self-reported measures, didn't let us control the conditions under which the questionnaire was administered, nor the influence of parents on the answers given. We cannot ensure that the questionnaire was administered in a calm environment, without distraction, and that the teenagers' attention was focused on it. In addition, single-item measures we used for stress level or social support, perceived less precision than a validated multi-item scale. Concerning changes in the use of coping strategies, although significant increases and decreases were observed, it should be noted that the effects size are small. Moreover, it is important to note the low reliability of the items measuring the strategies of active coping, venting, denial, self-distraction and behavioral disengagement. Another limitation is that our sample was located in the Auvergne-Rhône-Alpes region, which has a low urban density. This means that our sample had more access to the outdoors and the countryside, which are a source of more well-being (47, 48). Thus, our results could only be generalized to adolescents who spent the confinement in rural areas. A final important consideration is participation in the prevention program, which is a significant confounding variable. The program is based on the acquisition of psychosocial skills. These skills allow to acquire the necessary competencies to face situations. It is therefore also possible that some of the observed results may be due to participation in this program.

To conclude, the shift in the use of coping strategies enabled teenagers to be less stressed and decreased their substance use during the lockdown situation. However, this decrease in stress, also due to removal from the stressful environment of school, made it a source of distress for adolescents to return to school (49, 50). We think that extending the implementation of school-based prevention program based on the development of psychosocial skills could help adolescents to face the return to school.

## Data Availability Statement

The original contributions presented in the study are included in the article/Supplementary Material, further inquiries can be directed to the corresponding authors.

## Ethics Statement

The studies involving human participants were reviewed and approved by ANSM Registration Number: 2019-A03131-56. Written informed consent to participate in this study was provided by the participants' legal guardian/next of kin.

## Author Contributions

FM, GB, and FT contributed to conception and design of the study. CB organized the database, performed the statistical analysis, and wrote the first draft of the manuscript. All authors contributed to manuscript revision, read, and approved the submitted version.

## Funding

This research was supported by IRESP's funding partners within the framework of the 2018 General call for projects—Prevention and Health Promotion component Reference number: LI-BROUSSE-AAP18-PREV-001.

## Conflict of Interest

The authors declare that the research was conducted in the absence of any commercial or financial relationships that could be construed as a potential conflict of interest.

## Publisher's Note

All claims expressed in this article are solely those of the authors and do not necessarily represent those of their affiliated organizations, or those of the publisher, the editors and the reviewers. Any product that may be evaluated in this article, or claim that may be made by its manufacturer, is not guaranteed or endorsed by the publisher.

## References

[1] Wang C Pan R Wan X Tan Y Xu L Ho CS. Immediate psychological responses and associated factors during the initial stage of the 2019 coronavirus disease (COVID-19) epidemic among the general population in China. Int J Environ Res Public Health. (2020) 113:311–2. 10.3390/ijerph17051729 32155789 PMC7084952

[2] Hawryluck L Gold ML Robinson S Pogorski S Galea S Styra R. Control and psychological effects of quarantine, Toronto, Canada. Emerg Infect Dis. (2004) 10:1206–12. 10.3201/eid1007.030703 15324539 PMC3323345

[3] Jiao WY Wang LN Liu J Fang SF Jiao FY Pettoello-Mantovani M. Behavioral and emotional disorders in children during the COVID-19 epidemic. J Pediatr. (2020) 221:264–6. 10.1016/j.jpeds.2020.03.013 32248989 PMC7127630

[4] Taha S Matheson K Cronin T. Intolerance of uncertainty, appraisals, coping, and anxiety: the case of the 2009 H1N1 pandemic. Br J Heal. (2014) 19:592–605. 10.1111/bjhp.12058 23834735

[5] Zhang Y Ma Z. Impact of the COVID-19 pandemic on mental health and quality of life among local residents in Liaoning Province, China: a cross-sectional study. Int J Environ Res Public Health. (2020) 17:1–2. 10.3390/ijerph17072381 32244498 PMC7177660

[6] Jung SJ Jun JY. Mental health and psychological intervention amid COVID-19 outbreak: perspectives from South Korea. Yonsei Med J. (2020) 61:2716272. 10.3349/ymj.2020.61.4.271 32233168 PMC7105405

[7] Flaudias V Zerhouni O Pereira B Cherpitel CJ Boudesseul J de Chazeron I. The early impact of the COVID-19 lockdown on stress and addictive behaviors in an alcohol consuming student population in France. Front Psychol. (2021) 12:628–31. 10.3389/fpsyt.2021.628631 33633612 PMC7900161

[8] Lee J. Mental health effects of school closures during COVID-19. Lancet Child Adolesc Heal. (2020) 4:421. 10.1016/S2352-4642(20)30109-7 32302537 PMC7156240

[9] Singh S Roy D Sinha K Parveen S Sharma G Joshi G. Impact of COVID-19 and lockdown on mental health of children and adolescents: a narrative review with recommendations. Psychiatry Res. (2020) 293:113429. 10.1016/j.psychres.2020.113429 32882598 PMC7444649

[10] Guessoum SB Lachal J Radjack R Carretier E Minassian S Benoit I. Adolescent psychiatric disorders during the COVID-19 pandemic and lockdown. Psychiatry Res. (2020) 291:113264. 10.1016/j.psychres.2020.113264 32622172 PMC7323662

[11] de Araújo LA Veloso CF Souza MC Azevedo JMC Tarro G. The potential impact of the COVID-19 pandemic on child growth and development: a systematic review. J Pediatr (Rio J). (2020) 97:369–77. 10.1016/j.jped.2020.08.008 32980318 PMC7510529

[12] Guo J Fu M Liu D Zhang B Wang X van IJzendoorn MH. Is the psychological impact of exposure to COVID-19 stronger in adolescents with pre-pandemic maltreatment experiences? A survey of rural Chinese adolescents. Child Abuse Negl. (2020) 110:104667. 10.1016/j.chiabu.2020.104667 32859393 PMC7440157

[13] Cohen S Hoberman HM. Positive events and social supports as buffers of life change stress. J Appl Soc Psychol. (1983) 13:99–125. 10.1111/j.1559-1816.1983.tb02325.x

[14] Larocco JM House JS French J. A model of mental health, life events and social supports applicable to gênerai populations. J Health Soc Behav. (1980) 22:324–36. 10.2307/2136675

[15] Tousignant M. Soutien social et santé mentale : une revue de la littérature. Sci Soc Sante. (1988) 6:77–106. 10.3406/sosan.1988.1087

[16] Andersen HS Sestoft D Lillebæk T. A longitudinal study of prisoners onremand: psychiatric prevalence, incidence and psychopathology in solitaryvs. nonvsolitary confinement. Acta Psychiatr Scand. (2000) 102:19–25. 10.1034/j.1600-0447.2000.102001019.x 10892605

[17] Folkman S Lazarus RS. An analysis of coping in a middle aged community sample. J Health Soc Behav. (1980) 21:219–39. 10.2307/2136617 7410799

[18] Lazarus RS Folkman S. Stress, Appraisal and Coping. New York, NY : Springer (1984).

[19] Mages NL Mendelson GA. Effects of cancer on patients'lives: a personological approach. In: Adler, editor. Health Psychology: A Handbook. San Francisco, CA: Jossey-Bass.

[20] Compas BE Connor-Smith JK Saltzman H Thomsen AH Wadsworth ME. Coping with stress during childhood and adolescence: problems, progress, and potential in theory and research. Psychol Bull. (2001) 127:87–127. 10.1037/0033-2909.127.1.87 11271757

[21] Schwarzer R Schwarzer C. A critical survey of coping instrument. In: Zeidner M, Endler NS, editors. Handbook of Coping: Theory, Research and Applications. New york, NY: Wiley. p. 107–32.

[22] Williams K McGillicuddy-De Lisi A. Coping strategies in adolescents. J Appl Dev Psychol. (2004) 20:537–49. 10.1016/S0193-3973(99)00025-8

[23] Muller L Spitz E. Évaluation multidimensionnelle du coping: validation du Brief COPE sur une population française. Encephale. (2003) 29:507–18. 15029085

[24] Dunkley DM Blankstein KR Halsall J Williams M Winkworth G. The relation between perfectionism and distress: hassles, coping, and perceived social support as mediators and moderators. J Couns Psychol. (2000) 47:437–53. 10.1037/0022-0167.47.4.437

[25] Sasaki M Yamasaki K. Stress coping and the adjustment process among university freshmen. Couns Psychol Q. (2007) 20:51–67. 10.1080/09515070701219943

[26] Pritchard ME Wilson GS Yamnitz B. What predicts adjustment among college students?: a longitudinal panel study. J Am Coll Heal. (2007) 56:15–21. 10.3200/JACH.56.1.15-22 17711821

[27] Vanderbruggen N Matthys F Van Laere S Zeeuws D Santermans L Van den Ameele S. Self-reported alcohol, tobacco, and cannabis use during COVID-19 lockdown measures: results from a web-based survey. Eur Addict Res. (2020) 26:309–15. 10.1159/000510822 32961535 PMC7573904

[28] Niedzwiedz CL Green MJ Benzeval M Campbell D Craig P Demou E. Mental health and health behaviours before and during the initial phase of the COVID-19 lockdown: longitudinal analyses of the UK Household Longitudinal Study. J Epidemiol Community Health. (2021) 75:820. 10.1101/2020.06.21.20136820 32978210 PMC7892383

[29] Gaume J Schmutz E Zobel F. Évolution du marché des stupéfiants et de la situation des usagers durant l' épidémie de Covid-19 Résultats de la première vague d' enquête auprès des patient-e-s de la Policlinique d' addictologie du CHUV. Lausanne: Centre hospitalier universitaire vau.

[30] Kessler RC Berglund P Demler O Jin R Merikangas KR Walters EE. Lifetime prevalence and age-of-onset distributions of DSM-IV disorders in the national comorbidity survey replication. Arch Gen Psychiatry. (2005) 62:593–602. 10.1001/archpsyc.62.6.593 15939837

[31] Spilka S Godeau E Le Nézet O Ehlinger V Janssen E Brissot A. Usages d'alcool, de tabac et de cannabis chez les adolescents du secondaire en 2018 (2019).

[32] Michel G Purper-Ouakil D Mouren-Siméoni MC. Facteurs de risques des conduites de consommation de substances psycho-actives à l'adolescence. Ann Med Psychol (Paris). (2001) 159:622–31. 10.1016/S0003-4487(01)00102-0

[33] Windle M. The difficult temperament in adolescence: associations with substance use, family support, and problem behaviors. J Clin Psychol. (1991) 47:310–315. 10.1002/1097-4679(199103)47:2<lt;310::AID-JCLP2270470219>gt;3.0.CO;2-U 2030139

[34] Huba GJ Bentler PM. The role of peer and adult models for drug taking at different stages in adolescence. J Youth Adolesc. (1980) 9:449–65. 10.1007/BF02087681 24318204

[35] Neighbors B Kempton FR. Co-occurrence of substance abuse with conduct, anxiety, and depression disorders in juvenile delinquents. Addict Behav. (1992) 17:3796386. 10.1016/0306-4603(92)90043-U 1502971

[36] Mosbach P Leventhal H. Peer group identification and smoking. J Abnorm Psychol. (1988) 97:238–45. 10.1037/0021-843X.97.2.238 3385077

[37] Kreeft PVD Wiborg G Galanti MR Siliquini R Bohm K Scatigna M. EU-Dap Study Group. “Unplugged”: a new European school programme against substance abuse. Drugs Educ Prev Policy. (2009) 16:167–81. 10.1080/09687630701731189

[38] Carver CS. You want to measure coping but protocole's too long: consider the Brief COPE. Int J Behav Med. (1997) 4:92–100. 10.1207/s15327558ijbm0401_6 16250744

[39] Pierceall EA Keim MC. Stress and coping strategies among community college students. Community Coll J Res Pract. (2007) 31:703–12. 10.1080/10668920600866579

[40] Esparbès-Pistre S Bergonnier-Dupuy G Cazenave-Tapie P. Le stress scolaire au collège et au lycée: différences entre filles et garçons. É*duc Francoph*. (2015) 43:87–112. 10.7202/1034487ar

[41] Jones RW. Gender-specific differences in the perceived antecedents of academic stress. Psychol Rep. (1993) 72:739–43. 10.2466/pr0.1993.72.3.739 8332674

[42] Verma S Gupta J. Some aspects of high academic stress and symptoms. J Personal Clin Stud. (1990) 6:7–12.

[43] Subrahmanyam S. Academic adjustment and scholastic attainment of secondary school children. J Res Ext. (1986) 22:159–96.9417173

[44] Zakari S Walburg V Chabrol H. Influence de la pression perçue par les lycéens français sur le stress scolaire. J thérapie Comport Cogn. (2008) 18:108–12. 10.1016/j.jtcc.2008.06.006

[45] Brailovskaia J Rohmann E Bierhoff HW Schillack H Margraf J. The relationship between daily stress, social support and facebook addiction disorder. Psychiatry Res. (2019) 276:167–74. 10.1016/j.psychres.2019.05.014 31096147

[46] Sapolsky RM. The influence of social hierarchy on primate health. Science. (2005) 308:648–52. 10.1126/science.1106477 15860617

[47] Manusset S. Impacts psycho-sociaux des espaces verts dans les espaces urbains. Dével Durable Territ. (2012) 3. 10.4000/developpementdurable.9389

[48] Rolland B Haesebaert F Zante E Benyamina A Haesebaert J Franck N. Global changes and factors of increase in caloric/salty food intake, screen use, and substance use during the early COVID-19 containment phase in the general population in France: survey study. JMIR Public Heal Surveill. (2020) 6:19630. 10.2196/preprints.19630 32589149 PMC7505683

[49] Waters L Allen K-A Arslan G. Stress-related growth in adolescents returning to school after COVID-19 school closure. Front Psychol. (2021) 12:643443. 10.3389/fpsyg.2021.643443 34093323 PMC8174561

[50] Capurso M Dennis J Salmi L Parrino C Mazzeschi C. Empowering children through school re-entry activities after the COVID-19 pandemic. J Contin Educ Health Prof. (2020) 1:64–82. 10.5334/cie.17 PMC11104315 38774523

