# Supplementary material for: Lockdown Impact on Stress, Coping Strategies, and Substance Use in Teenagers
Source: Front Psychiatry. 2022 Jan 21;12:790704. doi: 10.3389/fpsyt.2021.790704 (PMC8813749; doi:10.3389/fpsyt.2021.790704)
Supplement: Supplementary file 1 [file Data_Sheet_1.docx]

**LOCKDOWN IMPACT ON STRESS, COPING STRATEGIES AND SUBSTANCE USE iN TEENAGERS**

C. Bourduge^a,c^, F. Teissedre^a^, F. Morel^c^, V. Flaudias^c,d^, M. Izaute^a^, G. Brousse^b^

**Supplementary material**

1. Substance use measure
2. Stress perceived measure
3. Social support measure
4. **Table S1.** Dimensions of the Brief-COPE evaluated from 1 to 4 (Muller & Spitz, 2003).

**Substance use measure**

How many cigarettes have you ever smoked? (check the corresponding box)

|  | 0 | 1-2 | 3-5 | 6-9 | 10-19 | 20-29 | 30 ou + |
| --- | --- | --- | --- | --- | --- | --- | --- |
| During your life | ☐ | ☐ | ☐ | ☐ | ☐ | ☐ | ☐ |
| During last 12 months | ☐ | ☐ | ☐ | ☐ | ☐ | ☐ | ☐ |
| During last 30 days | ☐ | ☐ | ☐ | ☐ | ☐ | ☐ | ☐ |

*N.B. Check 1-2 if you have ever smoked on a cigarette even without smoking the whole thing.*

Now, how many drinks have you had?

|  | 0 | 1-2 | 3-5 | 6-9 | 10-19 | 20-29 | 30 ou + |
| --- | --- | --- | --- | --- | --- | --- | --- |
| During your life | ☐ | ☐ | ☐ | ☐ | ☐ | ☐ | ☐ |
| During last 12 months | ☐ | ☐ | ☐ | ☐ | ☐ | ☐ | ☐ |
| During last 30 days | ☐ | ☐ | ☐ | ☐ | ☐ | ☐ | ☐ |

*N.B. Check 1-2 if you have ever tasted alcohol even without drinking a whole glass.*

Finally, how often have you used cannabis?

|  | 0 | 1-2 | 3-5 | 6-9 | 10-19 | 20-29 | 30 ou + |
| --- | --- | --- | --- | --- | --- | --- | --- |
| During your life | ☐ | ☐ | ☐ | ☐ | ☐ | ☐ | ☐ |
| During last 12 months | ☐ | ☐ | ☐ | ☐ | ☐ | ☐ | ☐ |
| During last 30 days | ☐ | ☐ | ☐ | ☐ | ☐ | ☐ | ☐ |

*N.B. Check 1-2 if you have ever smoked on a joint even without smoking the whole thing.*

**Stress perceived measure**

How stressed were you during the lockdown?

1 = Not stressed at all __________ 10 = Extremely stressed

1 2 3 4 5 6 7 8 9 10

Are you usually a stressed person?

1 = Not at all stressed __________ 10 = Extremely stressed

1 2 3 4 5 6 7 8 9 10

**Social support measure**

Did you miss your friends during the lockdown?

1 = Not at all __________ 10 = A lot

1 2 3 4 5 6 7 8 9 10

Have you been able to stay in contact with your friends through social networks, online games etc.?

1 = Not at all __________ 10 = A lot

1 2 3 4 5 6 7 8 9 10

How much time per day did you spend online with your friends?

Time per day (in hours)
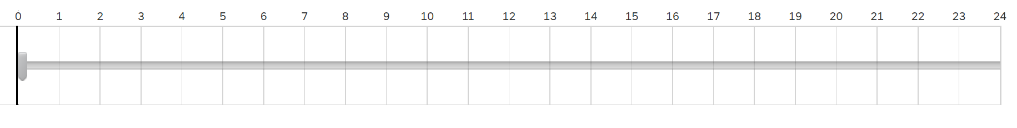


**Table S1.** Dimensions of the Brief-COPE evaluated from 1 to 4 (Muller & Spitz, 2003).

| ***Dimensions*** | ***Definition*** | ***Items*** |
| --- | --- | --- |
| Active coping | Process by which someone tries to eliminate the stressor or minimize its effects. | 2. I’ve been taking action to try to make the situation better.  20. I’ve been concentrating my efforts on doing something about the situation I’m in. |
| Planning | Thinking to organize a plan, steps to take and the best way to handle the problem. | 13. I’ve been trying to come up with a strategy about what to do.  24. I’ve been thinking hard about what steps to take. |
| Using instrumental support | Search for advice, assistance and/or information. | 10. I’ve been getting help and advice from other people.  19. I’ve been trying to get advice or help from other people about what to do. |
| Using emotional support | Rely on moral support, sympathy or understanding. | 5. I’ve been getting emotional support from others.  14. I’ve been getting comfort and understanding from someone. |
| Venting | Focus on emotional distress and express emotions. | 9. I’ve been saying things to let my unpleasant feelings escape.  18. I’ve been expressing my negative feelings. |
| Behavioral disengagement | Reduction of efforts to cope with the stressor, abandonment of any attempt to achieve the goals on which it interferes. | 6. I’ve been giving up trying to deal with it.  15. I’ve been giving up attempting to cope. |
| Self-distraction | Aims to divert thoughts related to the stressor or the goal with which it interferes. | 1. I’ve been turning to work or other activities to take my mind off things.  17. I’ve been doing something to think about it less, such as going to movies, watching TV, reading, daydreaming, sleeping, or shopping. |
| Self-blame | Blaming oneself. Is frequently associated with feelings of guilt. | 12. I’ve been criticizing myself.  25. I’ve been blaming myself for things that happened. |
| Positive reframing | Aims to manage emotional distress rather than fight the stressor. | 11. I’ve been trying to see it in a different light, to make it seem more positive.  26. I’ve been looking for something good in what’s happening. |
| Humor | Not taking the situation seriously to avoid being overwhelmed by emotions. | 16. I’ve been making jokes about it.  28. I’ve been making fun of the situation. |
| Denial | Refusing to believe that the stressor exists or attempting to act on the belief that the stressor is not real. | 3. I’ve been saying to myself “this isn’t real”.  21. I’ve been refusing to believe that it’s happened. |
| Acceptance | Accept the reality of a stressful situation. | 8. I’ve been accepting the reality of the fact that it’s happened.  23. I’ve been learning to live with it. |
| Religion | Reasons that may indicate that a person is turning to religion. | 7. I’ve been trying to find comfort in my religion or spiritual beliefs.  27. I’ve been praying or meditating. |
| Substance use | Use of alcohol, medication or drugs. | 4. I’ve been using alcohol or other drugs to make myself feel better.  22. I’ve been using alcohol or other drugs to help me get through it. |
